# Development of an AMBER-Compatible Force Field for Gold Nanoclusters Protected by *N*‑Heterocyclic Carbenes

**DOI:** 10.1021/acs.jctc.5c00945

**Published:** 2025-09-09

**Authors:** María Francisca Matus, Maryam Sabooni Asre Hazer, Sami Malola, Hannu Häkkinen

**Affiliations:** † Department of Physics, Nanoscience Center, 4168 University of Jyväskylä, Jyväskylä FI 40014, Finland; ‡ Department of Chemistry, Nanoscience Center, 4168 University of Jyväskylä, Jyväskylä FI 40014, Finland; § Carbon to Metal Coating Institute, Queen’s University, Kingston, Ontario K7L 3N6, Canada

## Abstract

*N*-Heterocyclic carbene (NHC)-protected gold nanoclusters (AuNCs) have emerged as promising candidates for biomedical applications due to their high stability and strong photoluminescence. However, their integration into atomistic molecular dynamics (MD) simulations, which facilitates an understanding of their behavior in biological environments, has been hindered by the lack of reliable force field parameters. Here, we present a new set of parameters for classical MD simulations of NHC-protected AuNCs, fully compatible with the AMBER force field. Validated through density functional theory (DFT)-based analyses and applied in a case study correlating ligand rigidity to optical properties, this work opens the door to predictive modeling and rational design of NHC-protected AuNCs for biomedical uses.

## Graphical abstract

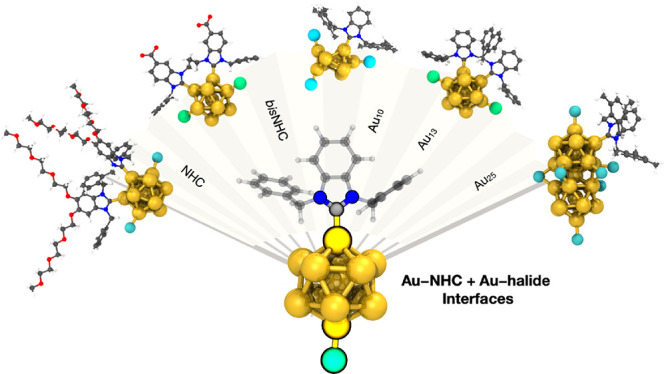

## 1 Introduction

*N*-Heterocyclic carbene (NHC)-protected gold nanoclusters (AuNCs) have gained significant attention in recent years due to their exceptional stability, tunable photophysical properties, and strong photoluminescence,^1–4^ making them ideal candidates for biomedical applications, including their use as imaging agents and as effective photosensitizers for photodynamic therapy.^1,3,5,6^ Recent efforts have led to the development of highly luminescent, water-soluble NHC-protected AuNCs with excellent chemical and thermal stability in biological media,^3,7^ thereby enhancing their potential for effective use *in vivo.* Despite these advancements, the exploration of NHC-protected AuNCs in biomedical contexts remains primarily experimental, with limited computational studies to predict and understand their behavior. Density functional theory (DFT) has proven to be a powerful tool for understanding the structure and properties of NHC-protected AuNCs, even offering predictive insights when experimental crystal structures are partially resolved or simply unavailable.^2,8–10^ However, to investigate the dynamic behavior of these nanoclusters and their interactions within complex biological environments, alternative computational approaches, such as molecular dynamics (MD) simulations, are more appropriate.^11–13^

To date, various classical and reactive force fields have been employed in MD simulations of different gold-based nanostructures.^14–20^ However, existing classical force fields are primarily designed to model bulk surfaces, colloidal nanoparticles, or thiolate-protected nanoclusters,^14,16,18,21^ resulting in limited applicability for systems with different compositions or architectures, such as atomically precise AuNCs stabilized by NHC ligands. Reactive force fields, such as ReaxFF, have shown promise due to their ability to model bond formation and breaking across a range of gold-based systems.^15,17,20,22^ However, their complexity, extensive parametrization, and incompatibility with standard biomolecular force fields present significant challenges for their integration into simulations of biologically relevant environments.^23^ On the other hand, hybrid quantum mechanics/molecular mechanics (QM/MM) approaches offer an alternative with higher accuracy and better transferability, but their high computational cost restricts access to long timescales and large systems.^24–26^ As a result, a critical gap remains in computational tools that can efficiently and accurately model the dynamic behavior of NHC-protected AuNCs, particularly within complex biological settings.

To address this gap, we introduce a new set of force field parameters specifically designed for classical MD simulations of NHC-protected AuNCs, developed to be fully compatible with the AMBER force field. This compatibility is a key advantage, as it enables seamless integration of nanocluster structures into established simulation frameworks involving a wide range of biomolecules―proteins, nucleic acids, lipids, and carbohydrates―for which AMBER has been extensively validated.^27^ The parameters were derived for one reference structure consisting of an Au_13_ core protected by monodentate NHC ligands but can be applied to several structures featuring diverse ligand chemistry and metal core geometry, as revealed by DFT-based MD simulations. Finally, a case study examining how the rigidity of the ligand layer impacts the photoluminescence of two nearly identical nanoclusters illustrates the practical applicability of these new parameters. This development can help accelerate the rational design of NHC-protected AuNCs for targeted biomedical applications.

## 2 Results and Discussion

### 2.1 Data Set

The atomistic structures of eight NHC-protected AuNCs were obtained from previous studies^2,3,5,7,10,28^ and were selected to represent diverse features based on their ligand chemistry and metal core geometry (Figure 1). The different types of NHC ligands can be categorized into three main groups: (i) monodentate NHC and organo-soluble, (ii) bidentate NHC and organo-soluble, and (iii) bidentate NHC and water-soluble. Similarly, the halide ligands can be classified as either (i) chloride or (ii) bromide. Regarding the metal core, three main sizes and geometries were included: (i) icosahedral Au_13_, (ii) toroidal Au_10_, and (iii) biicosahedral Au_25_ (derived from the conversion of toroidal Au_10_ nanoclusters). Details about the composition of each structure studied here are provided in Table 1.

**Figure 1:**
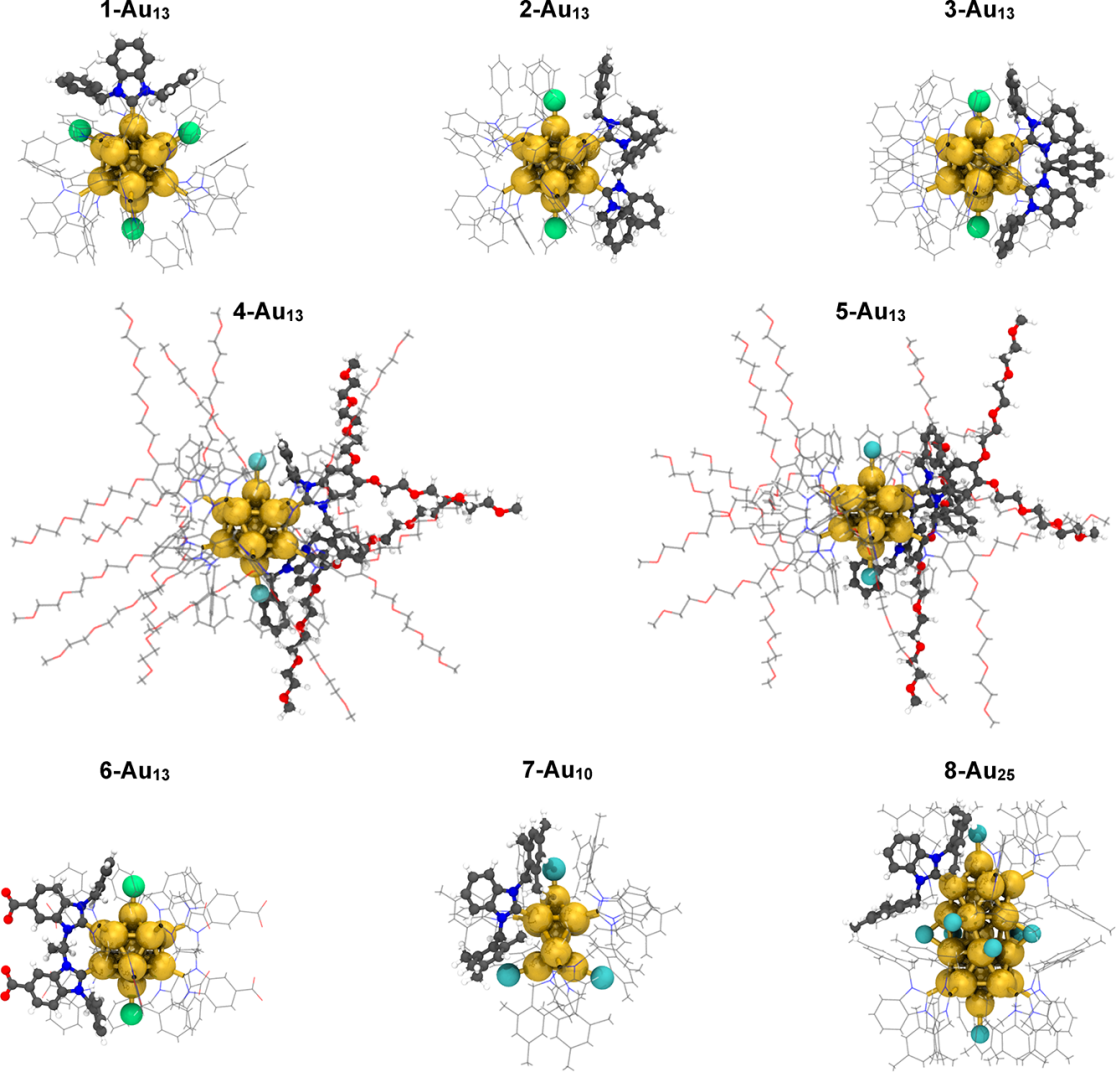
3D structures of the eight NHC-protected gold nanoclusters used in this study. Color code and representation: the metal core is depicted as golden spheres; halide ligands are shown as green spheres for chloride and cyan spheres for bromide; NHC ligands are represented as thin sticks, with carbon atoms in gray, nitrogen atoms in blue, oxygen atoms in red, and hydrogen atoms in white. In each structure, one NHC ligand is highlighted using a ball-and-stick representation to show whether it is monodentate or bidentate.

**Table 1:**
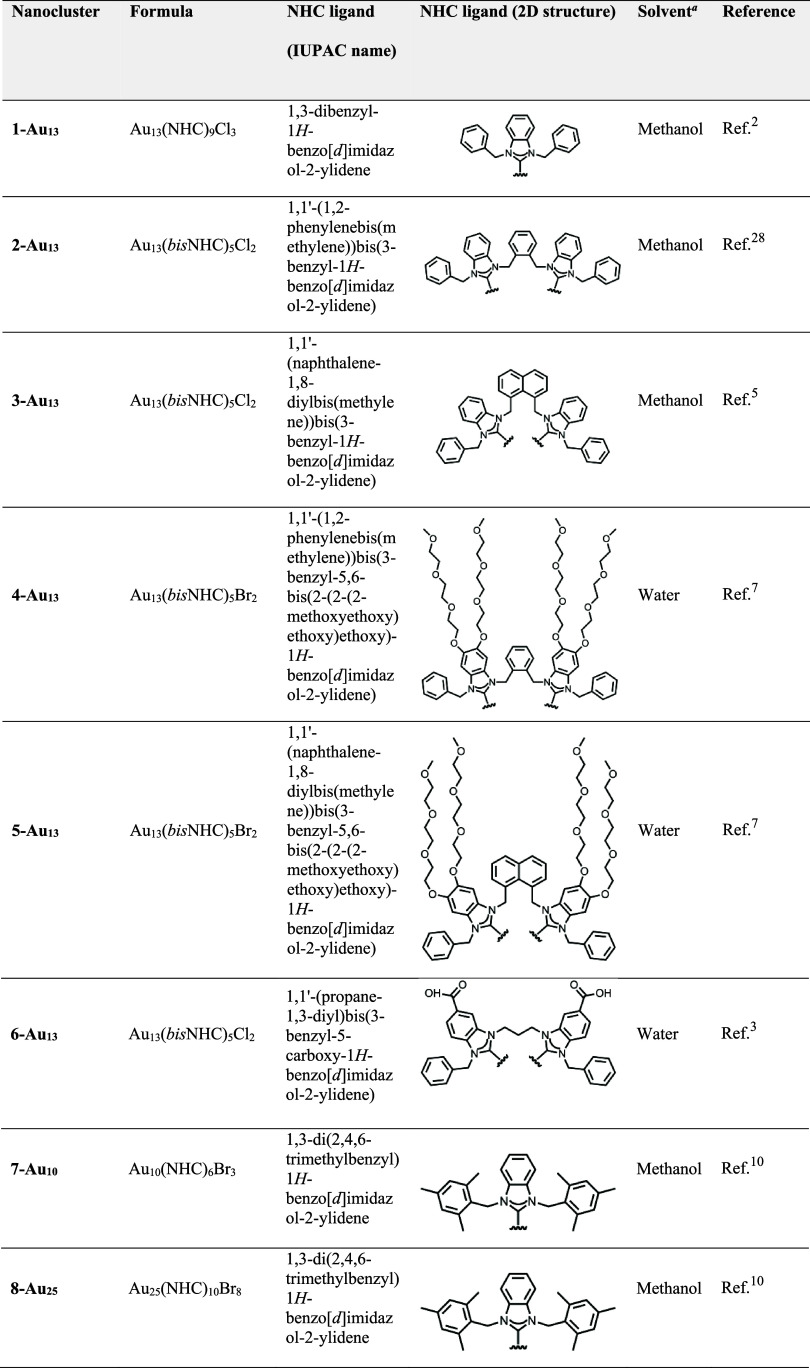
Composition of the Eight NHC-Protected Gold Nanoclusters Used in This Study

a Solvent used in classical MD simulations.

### 2.2 Bonded Parameters and Classical MD Simulations

We used **1-Au** **_13_** as the reference structure to derive the force constants and equilibrium values for a series of bonds and bond angles using DFT (see Section 3 for details). To maintain the integrity of the metal core structure, the metal–ligand interface was treated as covalently bound. As a first step, we determined the bonded parameters for the Au–C and Au–Cl bonds, as shown in Figure 2. Next, we obtained the equilibrium values for three new bond angles: Au–Au–C, Au–C–N, and Au–Au–Cl (Figure 3). By substituting the original halide ligand, we also obtained the parameters for the Au–Br bond and Au–Au–Br angle (Figures 2 and 3). The Au_13_ core features a highly symmetrical icosahedral structure, with one central Au atom surrounded by 12 others. Based on this arrangement, the Au–Au interactions within the metal core were described using a combination of bonded and nonbonded parameters. The bonded parameters were derived from vibrational frequencies previously calculated for the Au_13_ core of a thiolate-protected gold nanocluster^29^ and were applied to the 12 external Au atoms. Meanwhile, the nonbonded parameters were taken from the work of Heinz et al.^30^ and describe the interactions between the central Au atom and the Au atoms located at the vertices of the icosahedron.

**Figure 2:**
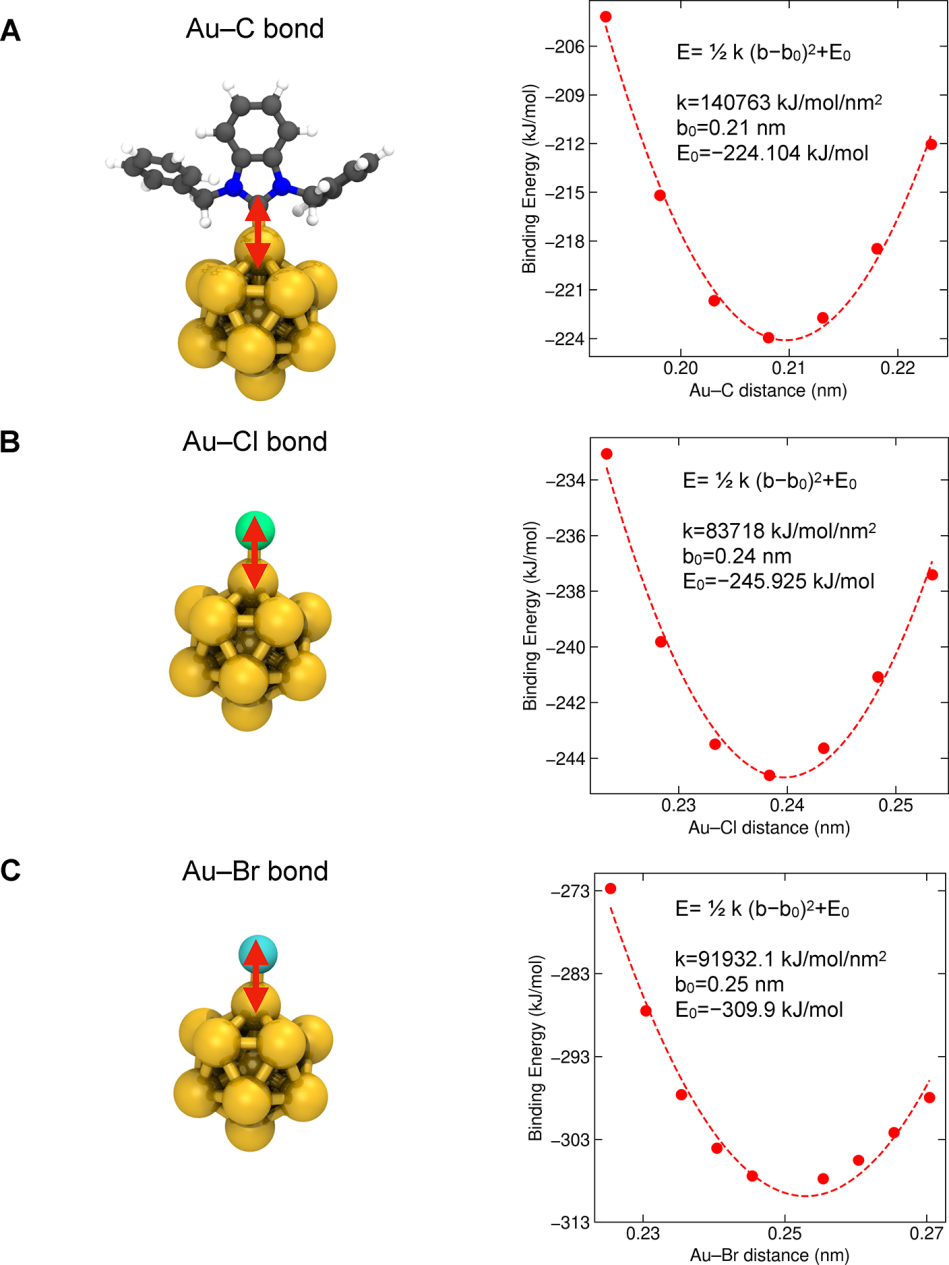
Force constants and equilibrium values for new bonds at the metal–ligand interface. Harmonic potential energy functions around the equilibrium bond distance *b* were calculated for (A) the Au–NHC interface and (B, C) the Au–halide interface. Color code and representation for the reference structure **1-Au** **_13_**: metal core, golden spheres; halide ligand, green sphere for chloride and cyan sphere for bromide; NHC ligand, ball-and-stick representation with carbon atoms in gray, nitrogen atoms in blue, and hydrogen atoms in white. For clarity, only one NHC or halide ligand is shown.

**Figure 3:**
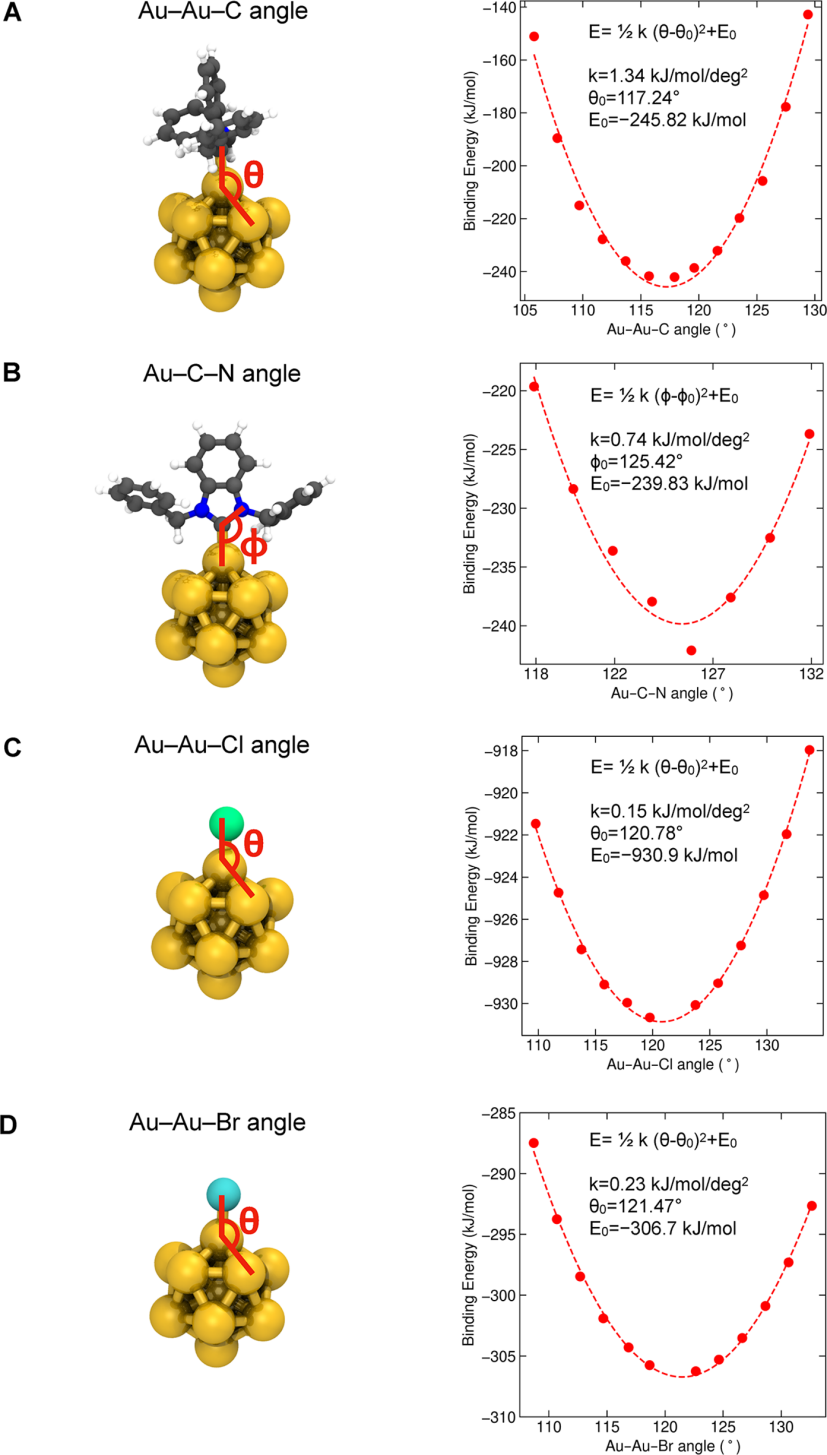
Force constants and equilibrium values for new angles at the metal–ligand interface. Harmonic potential energy functions around the equilibrium bond angle θ or ϕ were calculated for (A, B) the Au–NHC interface and (C, D) the Au–halide interface. Color code and representation for the reference structure **1-Au** **_13_**: metal core, golden spheres; halide ligand, green sphere for chloride and cyan sphere for bromide; NHC ligand, ball-and-stick representation with carbon atoms in gray, nitrogen atoms in blue, and hydrogen atoms in white. For clarity, only one NHC or halide ligand is shown.

The bond strength and length of Au–C were −224.104 kJ/mol and 0.21 nm, respectively, aligning with the values reported for the crystal structure.^2^ The harmonic force constant for the Au–C bond (see Table 2) is comparable to force constants in AMBER for standard organic systems and larger than the constant previously employed for the Au–S bond in thiolate-protected AuNCs,^18^ which reflects the stronger bonding and stiffer nature of the Au–NHC interaction.^31,32^ In contrast, the harmonic force constants for the Au–halide bonds, which exhibit less covalent stiffness, are an order of magnitude smaller than those found in AMBER for organic molecules (such as C–Cl or C–Br bonds). Additionally, the angle force constants calculated for the Au–Au–C and Au–C–N angles (see Table 2) are one order of magnitude bigger than those in AMBER for standard organic compounds, while the constants for Au–Au–halide angles (see Table 2) are similar to the values in AMBER (such as C–C–Cl and C–C–Br angles). The force constants for angle deformation typically reflect the stiffness of the bond angles; thus, the calculated values allow for a correct description of the varying flexibility in metal–organic and metal–inorganic environments within the nanoclusters.

**Table 2:** Compilation of All Bonded and Nonbonded Parameters Included in the Force Field for Classical MD Simulations

**bonded parameters**		
**bond**	***k*_b_** **(kJ mol^–1^ nm^–2^)**	***b*_0_ (nm)**
Au–C	140,763.0	0.20970
Au–Cl	83,718.0	0.23970
Au–Br	91,932.1	0.25000
Au–Au	42,278.3	0.30045

**angle**	***k***_**θ**_ **(kJ mol** ^**–1**^ **rad** ^**–2**^ **)**	**θ**_**0**_ **(degrees)**
Au–Au–C	2204.530	117.24
Au–C–N	1219.632	125.42
Au–Au–Cl	262.639	120.77
Au–Au–Br	377.544	121.47

**nonbonded parameters** ^a^		
**atom type**	**σ (nm)**	**ε** **(kJ mol** ^**–1**^ **)**
Au	0.262900	22.13300
Br	0.464693	0.245414

a Nonbonded parameters for Au and Br were taken from Heinz et al.,^30^ and from Joung and Cheatham,^33^ respectively.

After introducing new parameters to the force field (as listed in Table 2), each NHC-protected AuNC was simulated for 1 μs in a different solvent corresponding to the experimental conditions (see Table 1 and Section 3 for details), and no large fluctuations from the original geometry were observed (Figure S1). As in previous strategies,^18^ additional dihedral parameters for the metal–ligand interface were not needed, and only the parameters for bonds and angles were sufficient to simulate the behavior of the structures in solution without major structural disruptions.

Although the covalent network within the metal core imposes additional rigidity relative to purely nonbonded models, thereby preventing any deformation or transition of different symmetries in the metal core, our description remains valuable for exploring the dynamics of the entire structures since the ligand flexibility and solvent effects are well-captured in our simulations. To determine whether the high stability of the structures in the solvent is realistic and to demonstrate that this new set of parameters is suitable for describing the dynamics of NHC-protected AuNCs with varying ligand chemistry, core size, and shapes, we conducted DFT-based MD simulations and compared various observables.

### 2.3 Validation of Parameters

The dynamics of **1-Au** **_13_** (Au_13_ and monodentate NHC), **2-Au** **_13_** (Au_13_ and bidentate NHC), and **7-Au** **_10_** (core size other than Au_13_ and bromide as halide ligands) were studied over a picosecond timescale in vacuum using DFT-based MD simulations (see Section 3 for details), and the results were compared to those from classical MD simulations. In all cases, the bond distances and angles observed in classical MD simulations were consistent with those obtained from DFT-based MD simulations (see Table 3 and Figures S2–S11). The Au–Au–Cl angles monitored in **1-Au** **_13_** and **2-Au** **_13_** showed slightly more fluctuations in the DFT-based simulations (Figures S5 and S9), which may be attributed to the absence of the short-range solvent–halide coordination observed in the classical MD simulations (Figure S5), which restricts the halide movement; however, the mean values are in excellent agreement. The **7-Au** **_10_** nanocluster is the only structure in our data set with a nonsymmetrical metal core, resulting in different environments at the metal–ligand interface. The consistent results obtained from both classical and DFT-based MD simulations for this system suggest that our parameters are sufficiently accurate for classical MD simulations of NHC-protected AuNCs with different core geometries.

**Table 3:** Fluctuation of Bonds and Angles at the Metal–Ligand Interface (Mean ± SD) Obtained from Classical and DFT-Based MD Simulations^a^

**nanocluster**	**bond**	**Class-MD**	**DFT-MD**
**1-Au**_**13**_	Au–C	0.209 ± 0.001	0.209 ± 0.002
	Au–Cl	0.243 ± 0.003	0.240 ± 0.004
	**angle**	**Class-MD**	**DFT-MD**
	Au–Au–C	120.62 ± 0.61	122.45 ± 1.82
	Au–C–N	126.47 ± 2.50	126.58 ± 1.12
	Au–Au–Cl	124.88 ± 0.69	118.63 ± 1.76

**nanocluster**	**bond**	**Class-MD**	**DFT-MD**
**2-Au**_**13**_	Au–C	0.209 ± 0.001	0.209 ± 0.001
	Au–Cl	0.241 ± 0.003	0.236 ± 0.003
	**angle**	**Class-MD**	**DFT-MD**
	Au–Au–C	120.33 ± 0.95	121.87 ± 0.92
	Au–C–N	124.98 ± 1.18	126.34 ± 0.84
	Au–Au–Cl	122.61 ± 1.78	119.07 ± 1.60

**nanocluster**	**bond**	**Class-MD**	**DFT-MD**
**7-Au**_**10**_	Au–C	0.211 ± 0.002	0.208 ± 0.002
	Au–Br	0.252 ± 0.003	0.249 ± 0.003
	**angle**	**Class-MD**	**DFT-MD**
	Au–C–N	127.13 ± 1.72	126.63 ± 1.12

a All bond lengths are listed in nm and bond angles in degrees.

### 2.4 Case Study: The Influence of Ligand Layer Rigidity on the Quantum Yield of *bis*NHC-Protected AuNCs

To demonstrate the practical applicability of the obtained parameters, we selected two structures from our data set: **2-Au** **_13_** and **3-Au** **_13._** These structures share similar chemical and physical characteristics, including the size and shape of the metal core, the polarity and denticity of the NHC ligands, and the same halide ligand. The only difference between them is the presence of a π extension (the addition of an aromatic ring) at the linker of the bidentate NHC ligands (*bis*NHC). As shown in Table 1 (see 2D structures), the *bis*NHC ligands in **2-Au** **_13_** feature a benzyl linker, whereas in **3-Au** **_13_**, they have a naphthyl linker. This modification has recently demonstrated a significant effect on the photophysical properties of these nanoclusters, with a photoluminescence quantum yield (PLQY) increasing from 23 to 62% after the π extension.^5^ Although some assumptions related the differing fluorescence of the nanoclusters to the rigidity of their ligand layer, all observations were made with solid-state structures,^5^ which may differ substantially from the dynamics observed in solution. Therefore, we hypothesized that the rigidity of the ligand layer could be more thoroughly investigated through atomistic classical MD simulations in solution. This approach could help explain the increased PLQY observed in **3-Au** **_13._**

We collected data from three replicas of 1 μs for each structure and first compared the root-mean-square deviation (RMSD) and length of each *bis*NHC ligand over the simulated time. As shown in Figure 4A, the structural deviations of *bis*NHC ligands with the benzyl linker (hereinafter referred to as *bis*NHC^benzyl^) are higher than those with the naphthyl linker (denoted as *bis*NHC^naphthyl^). In the case of **2-Au** **_13_**, the RMSD values of *bis*NHC^benzyl^ ligands varied independently. Some ligands displayed slightly different conformations with respect to the initial structure, such as *bis*NHC-4 (RMSD of ∼0.15 nm), while others, like *bis*NHC-1 and *bis*NHC-5, exhibited more significant deviations (RMSD values >0.3 nm). In contrast, the RMSD of *bis*NHC^naphthyl^ ligands in **3-Au** **_13_** showed even movement, with average values around 0.32 nm for all of them. This result indicates a difference in the mobility of the ligand layer of both nanoclusters, which correlates with variations in ligand length. By measuring the distance between the outermost hydrogen atoms in the benzyl wingtip groups of each ligand (Figure 4B), we also observed independent variations in the lengths of *bis*NHC^benzyl^ ligands, ranging from 1.4 to 1.8 nm. In comparison, the structural movement of *bis*NHC^naphthyl^ ligands was consistent, resulting in minimal length fluctuations (from 1.56 to 1.58 nm on average).

**Figure 4:**
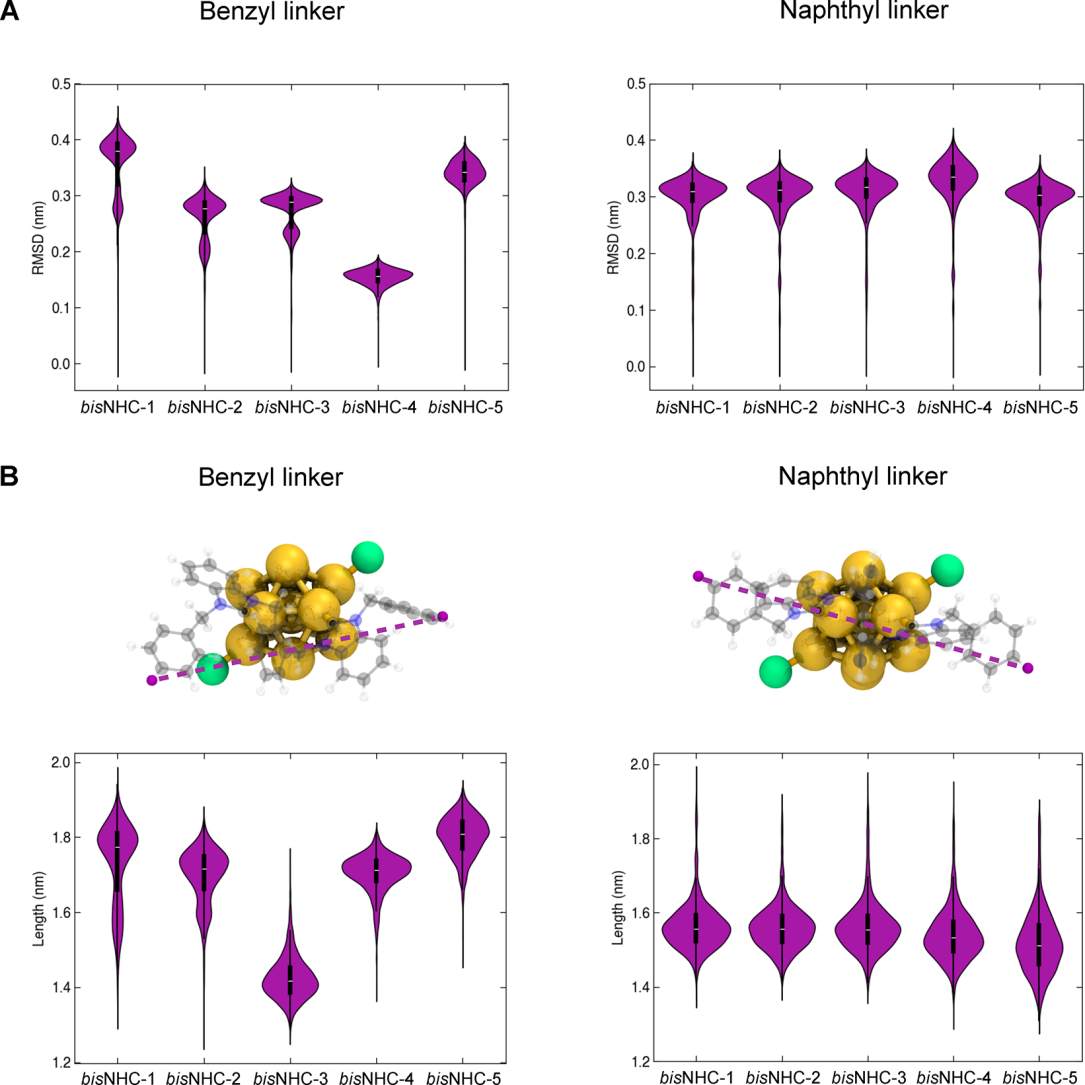
Structural deviations of *bis*NHC ligands in **2-Au_13_** and **3-Au_13_** nanoclusters. (A) Distribution of root-mean-square-deviation (RMSD) values for the heavy atoms of *bis*NHC ligands in **2-Au_13_** (benzyl linker) and **3-Au_13_** (naphthyl linker) nanoclusters. RMSD values were calculated with respect to the initial structure of the simulation (time = 0). (B) Upper panels: 3D representation of the nanoclusters with one *bis*NHC highlighted in a semitransparent ball-and-stick representation (Au in gold, Cl^–^ in green, C in gray, N in blue, and H in white). The dashed purple line indicates the interatomic distance used to quantify the ligand length. Lower panels: distribution of *bis*NHC ligand lengths in **2-Au_13_** (benzyl linker) and **3-Au_13_** (naphthyl linker) nanoclusters. Each plot shows the mean values calculated from three independent 1 μs molecular dynamics simulation replicas.

To analyze in detail the role of the linker in the mobility and stability of the ligand layers in these structurally similar nanoclusters, we monitored fluctuations in two internal dihedral angles that describe the *bis*NHC ligand flipping. The first dihedral angle (C^Au^–C^bridge^–C^Au^–C^bridge^) is formed between the carbon atoms in the benzimidazole units connected to gold and the carbon atoms in the methylene bridge that links the benzimidazole unit to the benzyl or naphthyl linker (see Figure 5). The second dihedral angle (C^wing^–C^Au^–C^Au^–C^wing^) is created between the outermost carbon atoms of the benzyl wingtip groups and the carbon atoms in the benzimidazole units connected to gold (see Figure 6).

**Figure 5:**
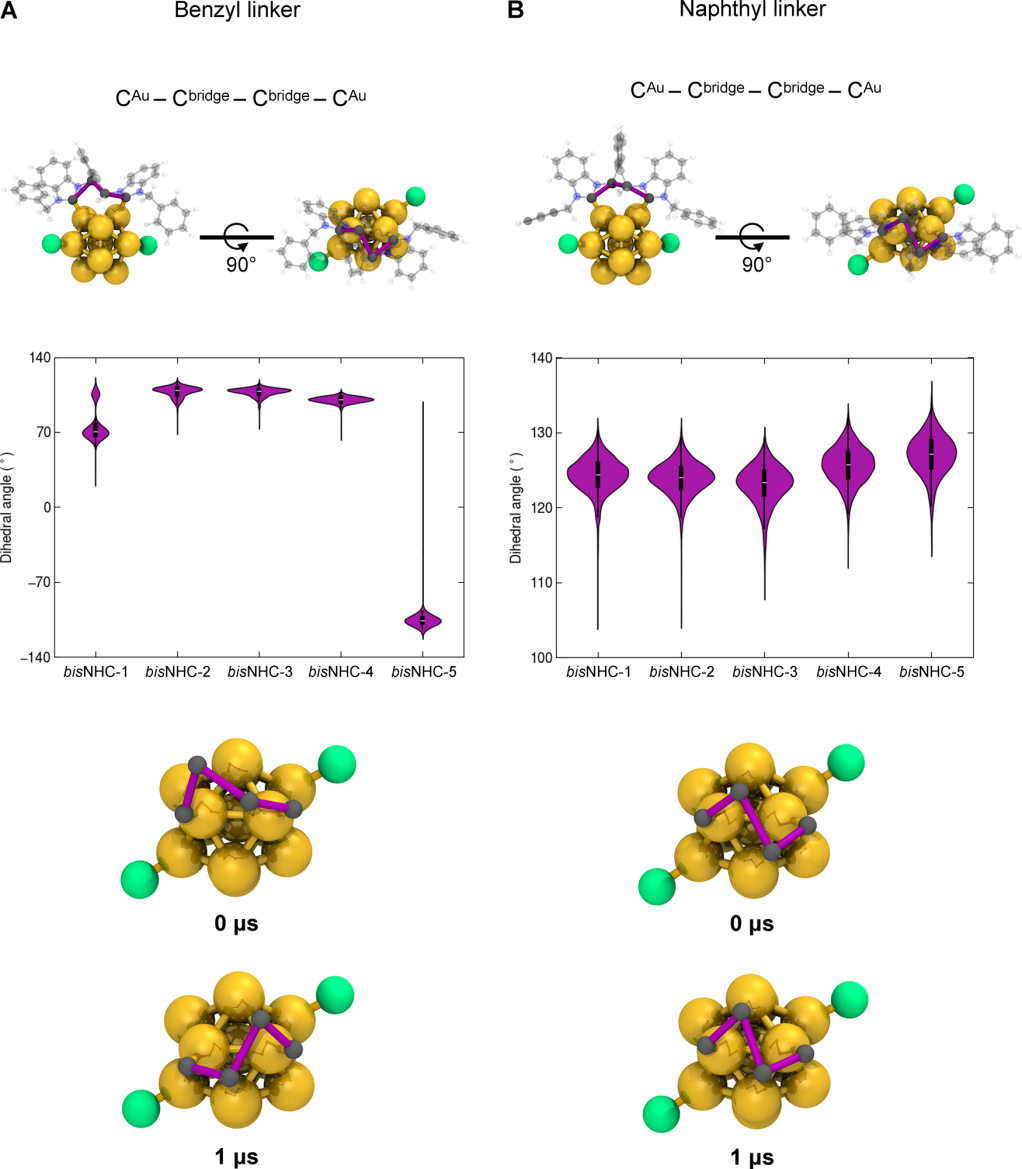
Conformational dynamics of *bis*NHC ligands in **2-Au**_**13**_ and **3-Au**_**13**_ nanoclusters analyzed by a short internal dihedral angle. Internal dihedral angle fluctuations were analyzed for (A) *bis*NHC ligands with a benzyl linker in the **2-Au** **_13_** nanocluster and (B) *bis*NHC ligands with a naphthyl linker in the **3-Au** **_13_** nanocluster. Upper panels: representative 3D structures of the nanoclusters viewed from two orthogonal perspectives, with one *bis*NHC shown in a semitransparent ball-and-stick representation (Au in gold, Cl^–^ in green, C in gray, N in blue, and H in white). The highlighted purple sticks trace four consecutive carbon atoms (C^Au^–C^bridge^–C^Au^–C^bridge^) within the linker, which define the dihedral angle used to describe internal torsional dynamics. Middle panels: violin plots showing the distribution of dihedral angle values for each *bis*NHC ligand across the 1 μs molecular dynamics simulations. The data are averaged over three independent replicas. Lower panels: snapshots of the *bis*NHC-5 ligand at the beginning (0 μs) and end (1 μs) of the simulation, highlighting conformational changes associated with dihedral angle flipping. The same atoms used to define the dihedral angle (in purple sticks) are shown to emphasize the observed torsional motion.

**Figure 6:**
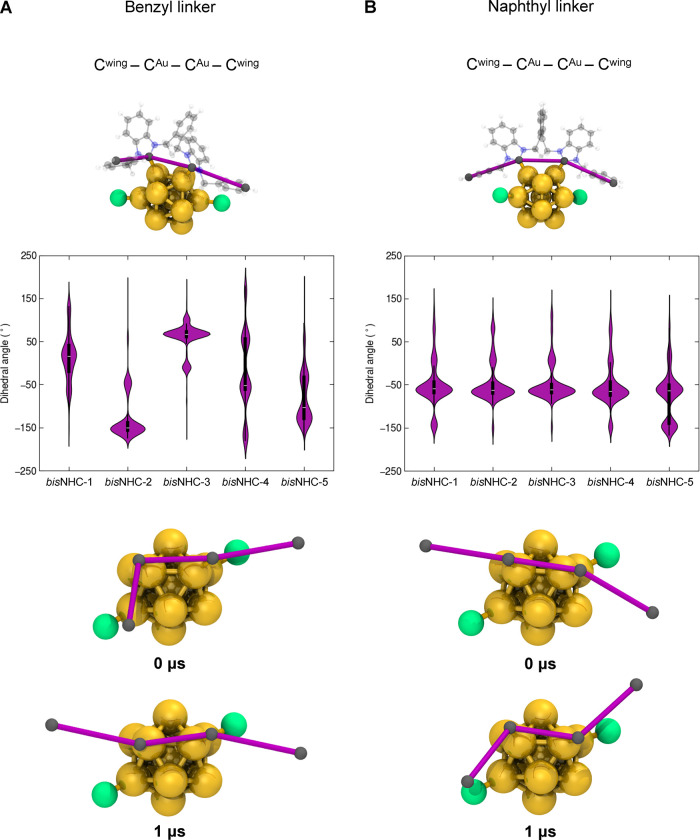
Conformational dynamics of *bis*NHC ligands in **2-Au**_**13**_ and **3-Au**_**13**_ nanoclusters analyzed by a long internal dihedral angle. Internal dihedral angle fluctuations were analyzed for (A) *bis*NHC ligands with a benzyl linker in the **2-Au** **_13_** nanocluster and (B) *bis*NHC ligands with a naphthyl linker in the **3-Au** **_13_** nanocluster. Upper panels: representative 3D structures of the nanoclusters with one *bis*NHC shown in a semitransparent ball-and-stick representation (Au in gold, Cl^–^ in green, C in gray, N in blue, and H in white). The highlighted purple sticks trace four consecutive carbon atoms (C^wing^–C^Au^–C^Au^–C^wing^) within the linker, which define the dihedral angle used to describe internal torsional dynamics. Middle panels: violin plots showing the distribution of dihedral angle values for each *bis*NHC ligand across the 1 μs molecular dynamics simulations. The data are averaged over three independent replicas. Lower panels: snapshots of the *bis*NHC-5 ligand at the beginning (0 μs) and end (1 μs) of the simulation, highlighting conformational changes associated with dihedral angle flipping. The same atoms used to define the dihedral angle (in purple sticks) are shown to emphasize the observed torsional motion.

As illustrated in Figure 5A and Figure S12, the first dihedral angle for *bis*NHC^benzyl^ ligands was consistently similar for *bis*NHC-2, *bis*NHC-3, and *bis*NHC-4 (ranging from 90 to 100°). In contrast, *bis*NHC-1 exhibited two populations at ∼100 and 70°, the latter due to a counterclockwise rotation observed in one of the three replicas and maintained for 80 ns. The most significant conformational change occurred with *bis*NHC-5, which showed a clear ligand flipping after 10 ns (see the initial and final snapshots in Figure 5A). On the other hand, no notable changes were detected in the *bis*NHC^naphthyl^ ligands, which maintained a relatively similar conformation throughout the MD trajectories (see Figure 5B and Figure S12). A similar trend was observed when monitoring the second dihedral angle over the simulated time for both structures. This angle, which includes atoms from the wingtip groups, reflects the rotation along the entire *bis*NHC ligands. The *bis*NHC^benzyl^ ligands exhibited several arrangements (see Figure 6A and Figure S13), including clockwise and counterclockwise rotations during the simulations. For instance, *bis*NHC-3 adopted an eclipsed conformation (see Figure S13), while the others predominantly displayed Z-conformations (positive values for *bis*NHC-1 and *bis*NHC-3 and negative values for *bis*NHC-2 and *bis*NHC-5). In contrast, the angle distributions of the *bis*NHC^naphthyl^ ligands were nearly identical, as shown in Figure 6B. The rotations of the ligands occurred simultaneously and contributed to the twisted geometry observed at the end of the simulations. These findings strongly suggest that the naphthyl linker enhances the rigidity of the ligand layer in **3-Au** **_13_**, thereby limiting the independent movement of the *bis*NHC ligands in solution and resulting in a higher PLQY. In all conformations observed, the benzimidazole units twist in equal and opposite directions around the linker, consistent with the previously reported crystal structure.^5^ This behavior sharply contrasts with the asymmetric twist found in **2-Au** **_13,_** which, in turn, allows for greater flexibility in the *bis*NHC ligands.

This case study illustrates how understanding the dynamics of the ligand layer through MD simulations provides key insights into the relationship between the structure and properties of NHC-protected AuNCs. These insights are essential for the rational design of nanoclusters with customized attributes for various applications, including sensing, diagnostics, and therapeutics.

## 3 Methods

### 3.1 Parametrization

Force constant parameters for bonds and bond angles at the metal–ligand interface were derived using DFT calculations with the GPAW software package,^34,35^ utilizing **1-Au** **_13_** as the reference structure (see Figure 1 and Table 1). All calculations were carried out using the Perdew–Burke–Ernzerhof (PBE) exchange-correlation functional^36^ and a real-space grid with a grid spacing of 0.2 Å. The atomic structure of **1-Au** **_13_** nanocluster was first optimized until the forces on atoms were below the optimization convergence criterion of 0.05 eV/Å. Then, a single representative monodentate NHC ligand or halide ligand was selected from the nanocluster surface to study the energy behavior around the equilibrium position. This was done by varying the bond distance from the ligand to the metal core by increments of 0.10 Å. A similar approach was used to determine the force constants for the bond angles (Au–Au–C and Au–C–N for the NHC ligand and Au–Au–Cl or Au–Au–Br for the halide ligand) by rotating the ligand at the metal core surface in increments of 2°. The axes of rotation were parallel to the metal core surface, with the rotation center fixed at the second atom in the specified angle (e.g., the carbon atom for the Au–C–N angle). Finally, in both cases, a harmonic function was fitted to the DFT energies to calculate the harmonic force constants.

### 3.2 Classical MD Simulations

The atomic structures of the eight NHC-protected gold nanoclusters used in this work were obtained from previous studies (see Figure 1 and Table 1) and optimized with DFT. The partial charges on the NHC atoms were derived with Ambertools16,^37^ using a model system consisting of two gold atoms connected to either two monodentate NHC ligands or one bidentate NHC ligand. The geometry of the Au_2_(NHC)_2_ or Au_2_(*bis*NHC)_1_ models was optimized at a B3LYP/LANL2DZ/W06 level of theory,^38^ and the resulting electron density was used to calculate the electrostatic potential according to the Merz–Singh–Kollman scheme^39,40^ using Gaussian 09.^41^ The atomic charges were fitted to the calculated potential through a two-stage restrained electrostatic potential (RESP) fitting procedure, constraining the charges of Au atoms to zero, as previously reported.^18,42^ This choice is supported by the computed Bader charges of gold atoms in **1-Au** **_13_**, which are close to zero,^2^ and enhances the transferability of the derived force field parameters across different NHC-protected AuNCs with varying sizes and geometries. GROMACS topologies for each NHC ligand were then generated using the ACPYPE code.^43^ Subsequently, each nanocluster was placed at the center of a periodic cubical box filled with water molecules (TIP3P model)^44^ or methanol (see Table 1), along with sufficient Na^+^ counterions to neutralize the system.^45^ Energy minimization was performed with the steepest descent algorithm, followed by a short two-step equilibration process. This included (1) a 10 ns simulation in the NVT ensemble at 300 K using a velocity-rescaling thermostat^46^ and (2) a 10 ns simulation in the NPT ensemble at 300 K and 1 bar pressure using a stochastic cell rescaling (C-rescale) barostat^47^ (isothermal compressibilities of water and methanol were 4.5 × 10^–5^ and 1.2 × 10^–4^ bar^–1^, respectively).^48^ During the equilibration phase, position restraints were applied to all nonhydrogen AuNC atoms. Afterward, the restraints were removed and 1 μs of production MD simulation was conducted while maintaining a temperature of 300 K and a pressure of 1 bar, with a time step of 2.0 fs. The electrostatic interactions were modeled using the particle-mesh Ewald (PME) method^49^ with a cutoff of 1.0 nm and a grid spacing of 0.12 nm, while the van der Waals interactions were modeled with Lennard–Jones potentials, truncated at 1.0 nm. For improved performance, the linear constraint solver (LINCS) algorithm was used to constrain the bond lengths to hydrogens.^50^ All MD simulations were performed with GROMACS 2022.^51^

### 3.3 DFT-Based MD Simulations

MD simulations based on DFT were performed on the optimized structures of **1-Au** **_13_**, **2-Au** **_13_**, and **7-Au** **_10_** nanoclusters (see Figure 1 and Table 1) by using the PBE exchange-correlation functional^36^ and the Langevin thermostat with a friction parameter of 0.01 fs^–1^. The atomic mass of all hydrogen atoms was replaced by the mass of deuterium to allow a simulation time step of 2.0 fs. Changing the masses of hydrogen affects the specific vibrational frequencies of bonds such as C–H. However, this change has a negligible impact on the overall dynamic behavior of the ligands, as well as on the dynamics of the Au–C and Au–Cl bonds at the metal–ligand interface. The total duration of each simulation run was 10 ps, which included a phase for heating the system to the target temperature of 300 K. Analysis was conducted after the system had reached thermal equilibration, specifically excluding the initial 3 ps. All DFT-based MD simulations were performed with GPAW.^34,35^

## 4 Conclusions

We have developed a new set of AMBER-compatible parameters specifically optimized for atomistic classical MD simulations of NHC-protected AuNCs of various sizes, shapes, and surface chemistries in solvent. In addition, the procedure used to parametrize the NHC ligands is versatile and can be effectively applied to a variety of other NHC ligands. As a result, parameters for NHC-protected AuNCs with new surface chemistries can be easily obtained without needing to change the existing metal core or metal–ligand interface parameters. This feature is especially attractive, for instance, for exploring tridentate NHC ligands (*tris*NHC) as protecting ligands, which have not yet been documented in the literature. These ligands may contribute to the formation of nanoclusters that exhibit not only well-defined structures but also enhanced photophysical properties, particularly beneficial for biomedical applications. Similarly, these parameters provide new opportunities for investigating a novel class of NHC-protected AuNCs with targeting motifs. Atomistic simulations of these functionalized nanoclusters in conjunction with their macromolecular receptors, such as proteins, can provide deeper insights into critical binding events and structural arrangements, thereby advancing the development of targeted therapies. Overall, these new parameters are well-suited to capture the dynamic behavior of NHC-protected AuNCs in the solvent and ready to be explored in complex biological systems, which is essential for realizing the potential of these nanostructures in diverse areas of biomedicine.

## Supplementary Material





## Notes

Additional data on MD simulations of NHC-protected gold nanoclusters (PDF)
Force field parameters and GROMACS topologies of NHC-protected gold nanoclusters (ZIP)
