# Supplementary material for: Development of an AMBER-Compatible Force Field for Gold Nanoclusters Protected by N‑Heterocyclic Carbenes
Source: J Chem Theory Comput. 2025 Sep 9;21(23):12121–32. doi: 10.1021/acs.jctc.5c00945 (PMC12874363; doi:10.1021/acs.jctc.5c00945)
Supplement: Supplementary file 1 [file ct5c00945_si_001.pdf]

Supporting Information

Development of an AMBER-Compatible Force Field for Gold Nanoclusters Protected by *N*-Heterocyclic Carbenes

María Francisca Matus,[†,§,*] Maryam Sabooni Asre Hazer,[†,§] Sami Malola,[†,§] Hannu Häkkinen[†,‡,§*]

†Department of Physics, Nanoscience Center, University of Jyväskylä, Jyväskylä, FI 40014, Finland.

‡Department of Chemistry, Nanoscience Center, University of Jyväskylä, Jyväskylä, FI 40014, Finland.

§Carbon to Metal Coating Institute, Queen's University, Kingston, Ontario K7L 3N6, Canada.

*Corresponding authors. E-mail: maria.f.matus-cortes@jyu.fi; hannu.j.hakkinen@jyu.fi

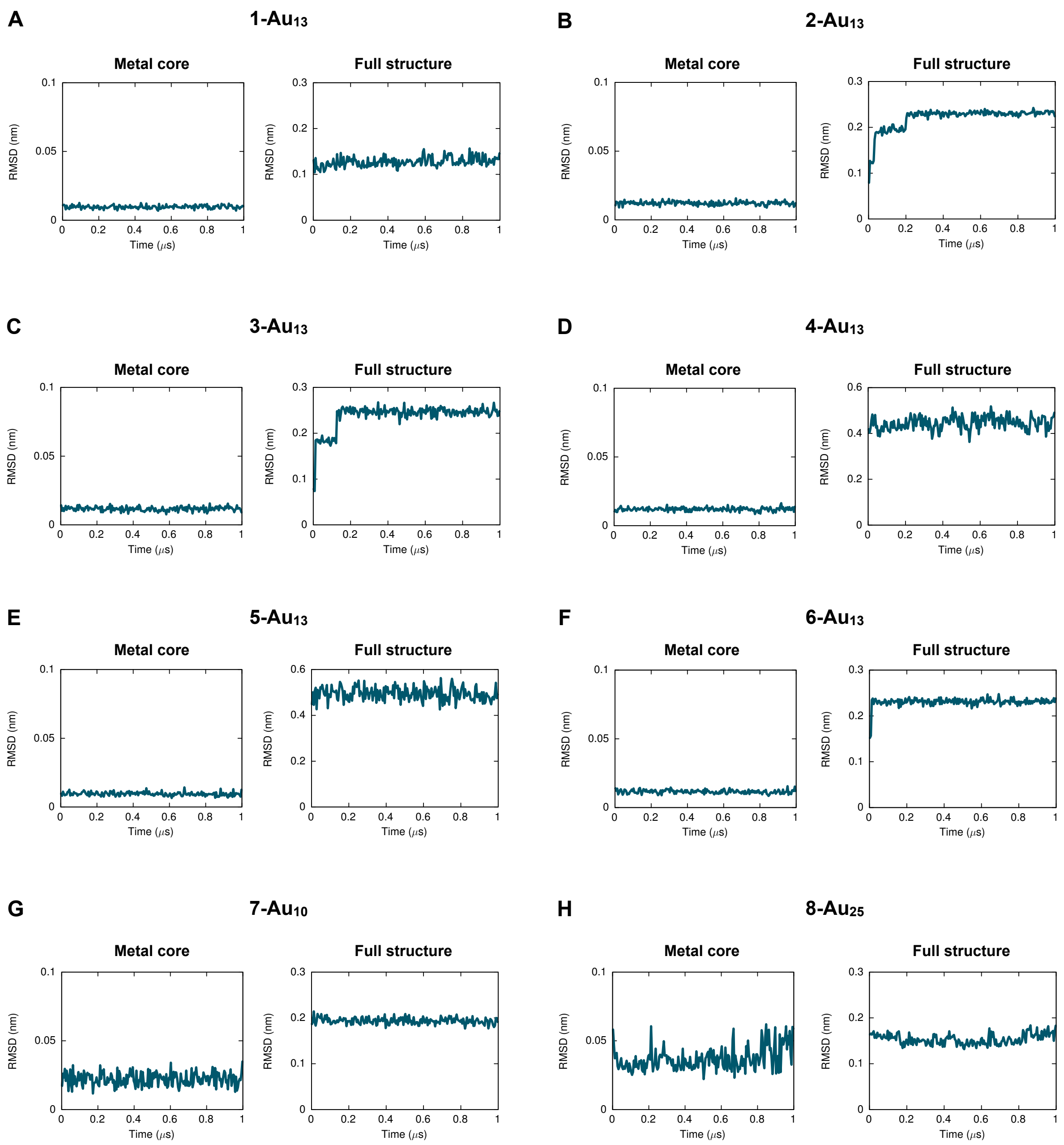

Figure S1. Root-mean-square deviation (RMSD) as a function of time during the MD simulations of the eight NHC-protected gold nanoclusters used in this study. RMSD values were calculated with respect to the initial structure of the simulation (time = 0).

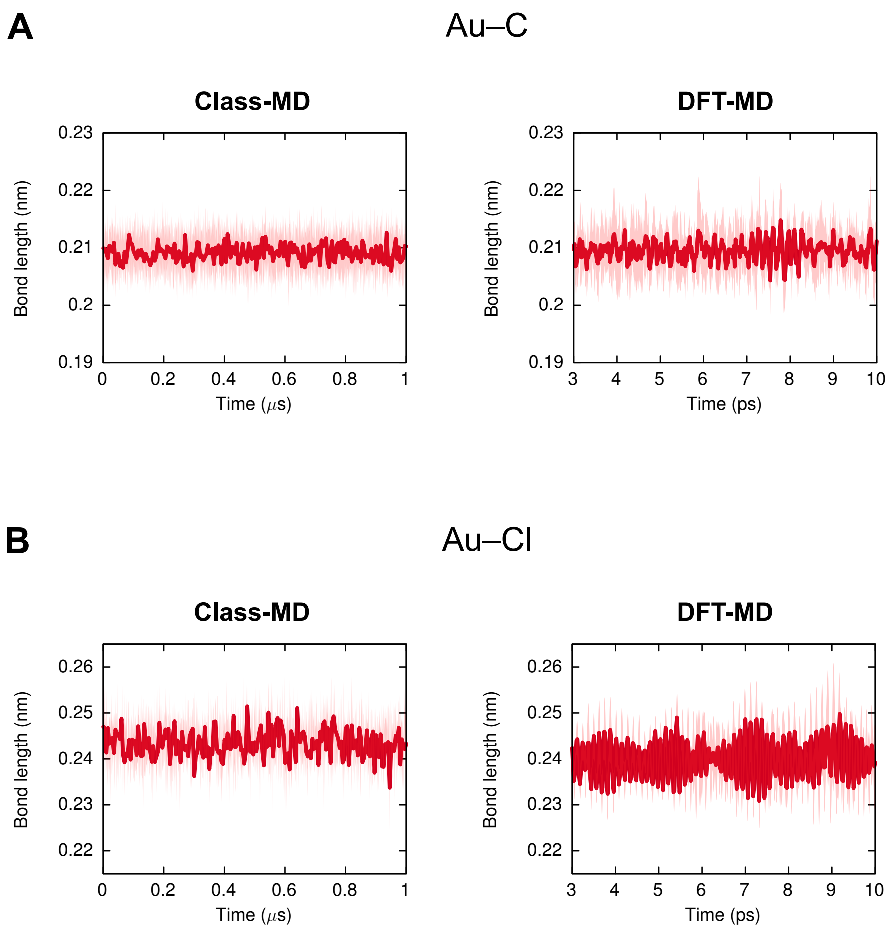

Figure S2. Fluctuation of Au−C and Au−Cl bond lengths as a function of time during the MD simulations of 1-Au_{13} nanocluster. (A) Each plot shows the mean bond length (thick line) ± standard deviation (shaded area) of 9 measurements per frame. (B) Each plot shows the mean bond length (thick line) ± standard deviation (shaded area) of 3 measurements per frame.

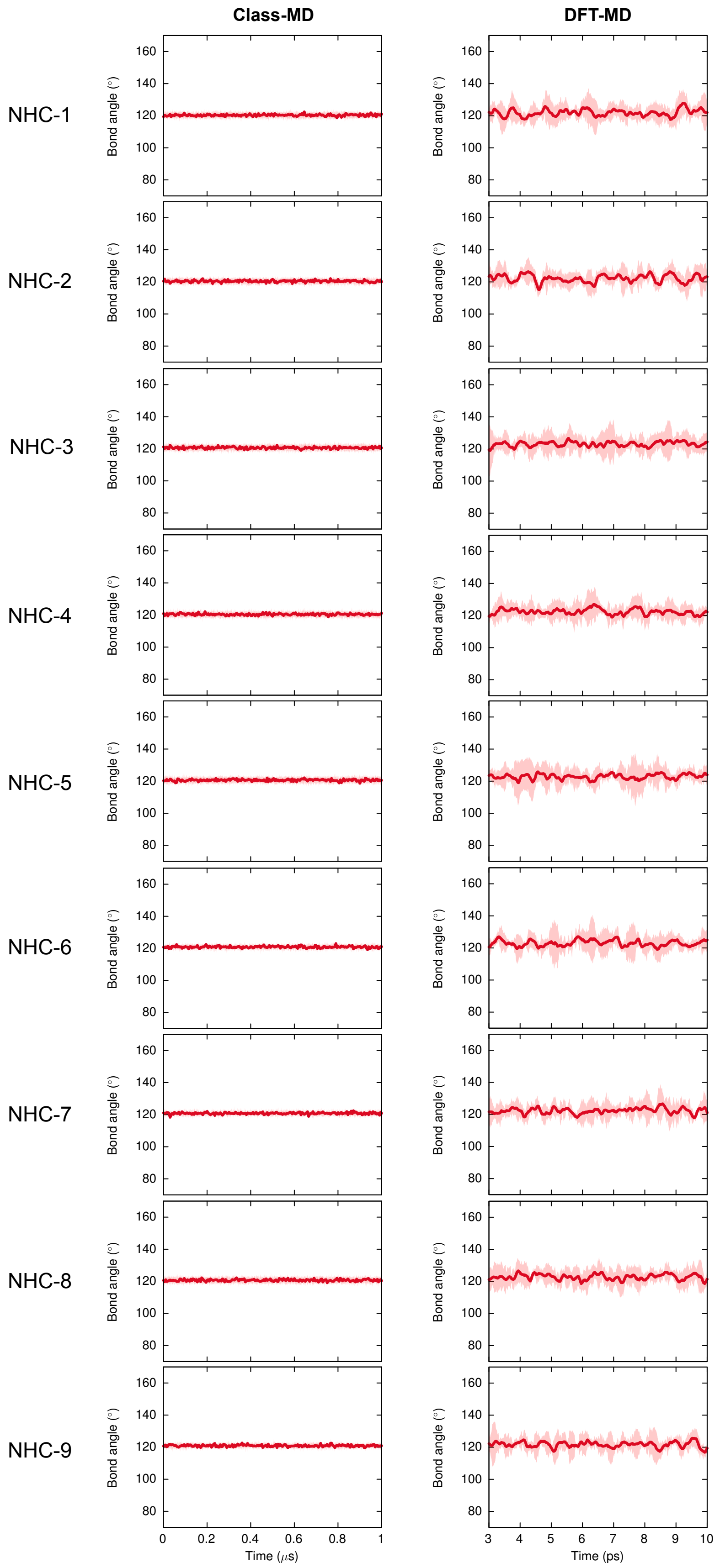

Figure S3. Fluctuation of Au−Au−C angle as a function of time during the MD simulations of 1-Au_{13} nanocluster. Each plot shows the mean bond angle (thick line) ± standard deviation (shaded area) of 5 measurements per frame.

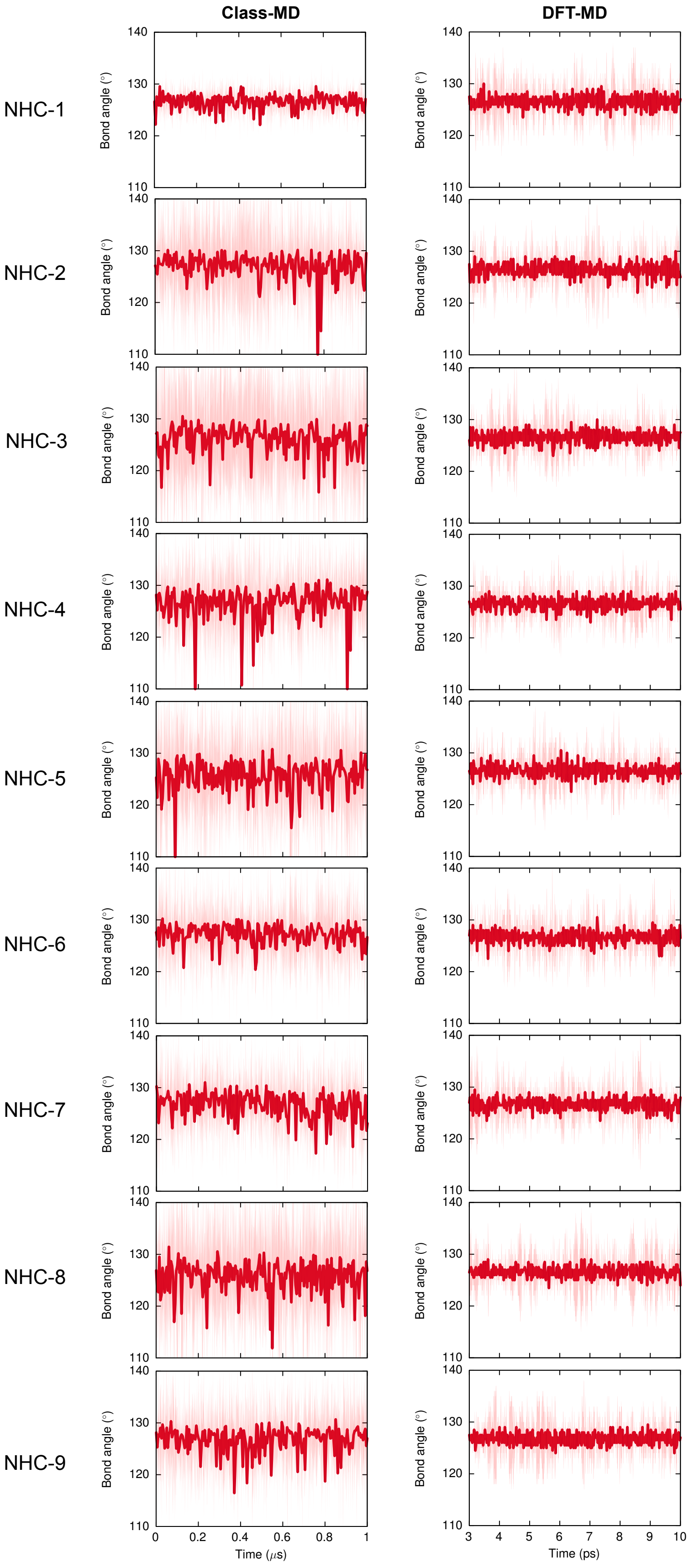

Figure S4. Fluctuation of Au−C−N angle as a function of time during the MD simulations of 1-Au_{13} nanocluster. Each plot shows the mean bond angle (thick line) ± standard deviation (shaded area) of 2 measurements per frame.

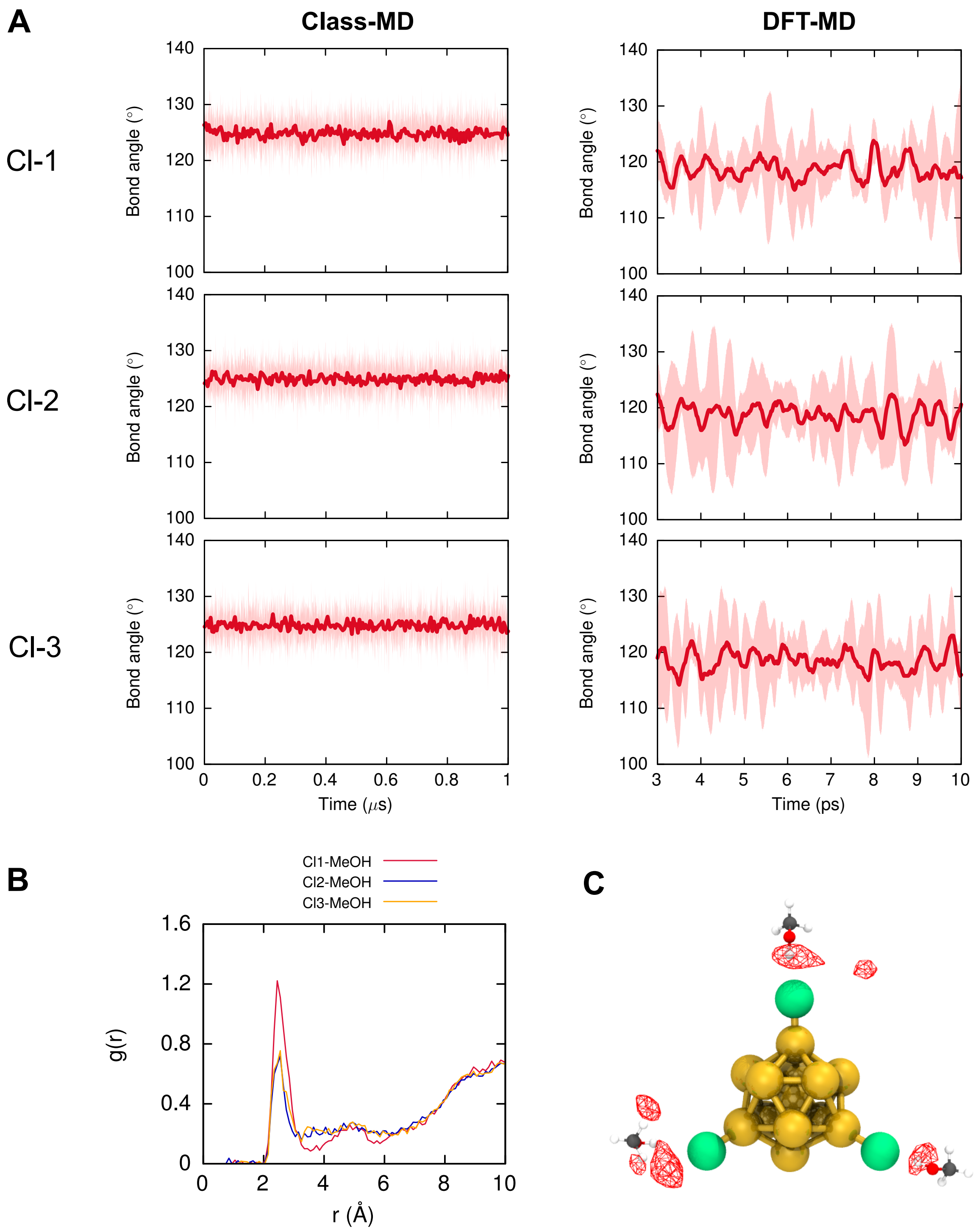

Figure S5. Fluctuation of Au−Au−Cl angle and halide−solvent interactions as a function of time during the MD simulations of 1-Au_{13} nanocluster. (A) Each plot shows the mean bond angle (thick line) ± standard deviation (shaded area) of 5 measurements per frame. (B) Radial distribution function (RDF) between the hydroxyl hydrogen atoms of methanol and the chloride ligands in the nanocluster. (C) Spatial density maps of methanol atoms (hydroxyl hydrogen) around the chloride ligands. One of the closest methanol molecules is shown using balls-and-sticks representation to illustrate the density maps shown in red wireframes. Color code: Au in gold, Cl^- in green, C in grey, O in red, H in white.

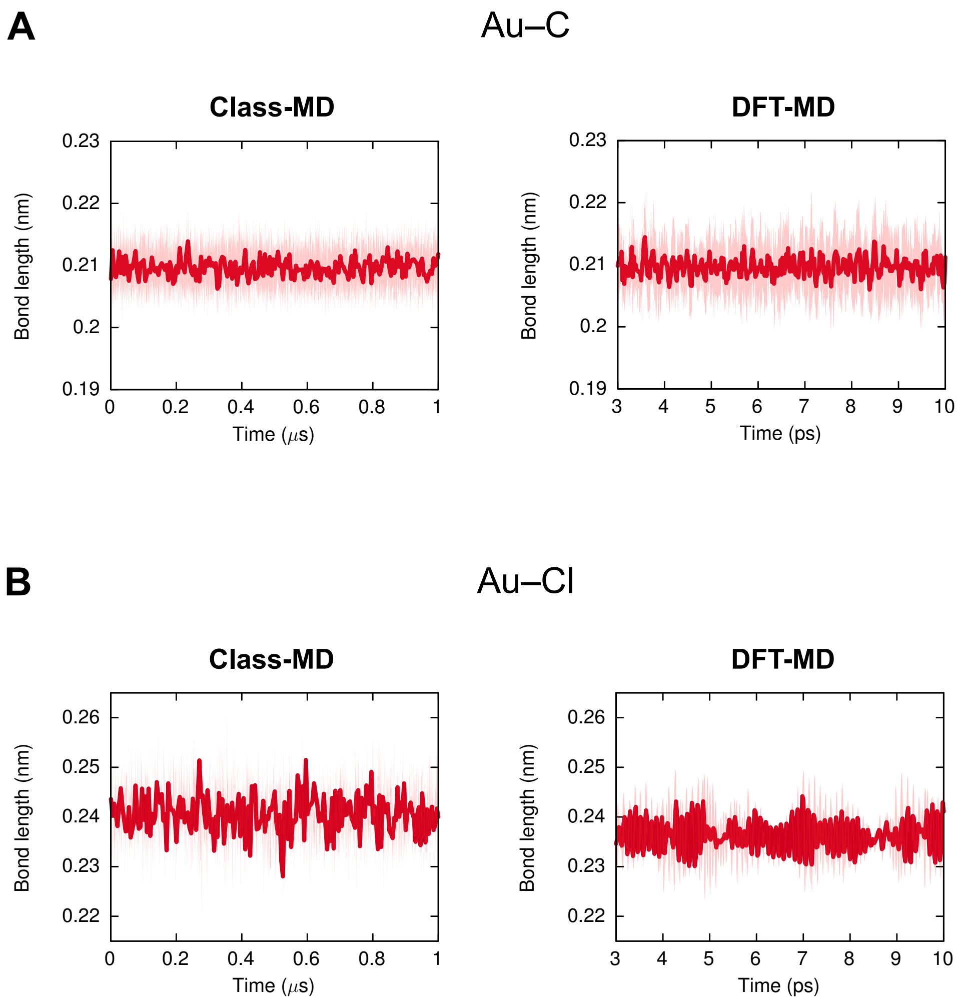

Figure S6. Fluctuation of Au−C and Au−Cl bond lengths as a function of time during the MD simulations of 2-Au_{13} nanocluster. (A) Each plot shows the mean bond length (thick line) ± standard deviation (shaded area) of 10 measurements per frame. (B) Each plot shows the mean bond length (thick line) ± standard deviation (shaded area) of 2 measurements per frame.

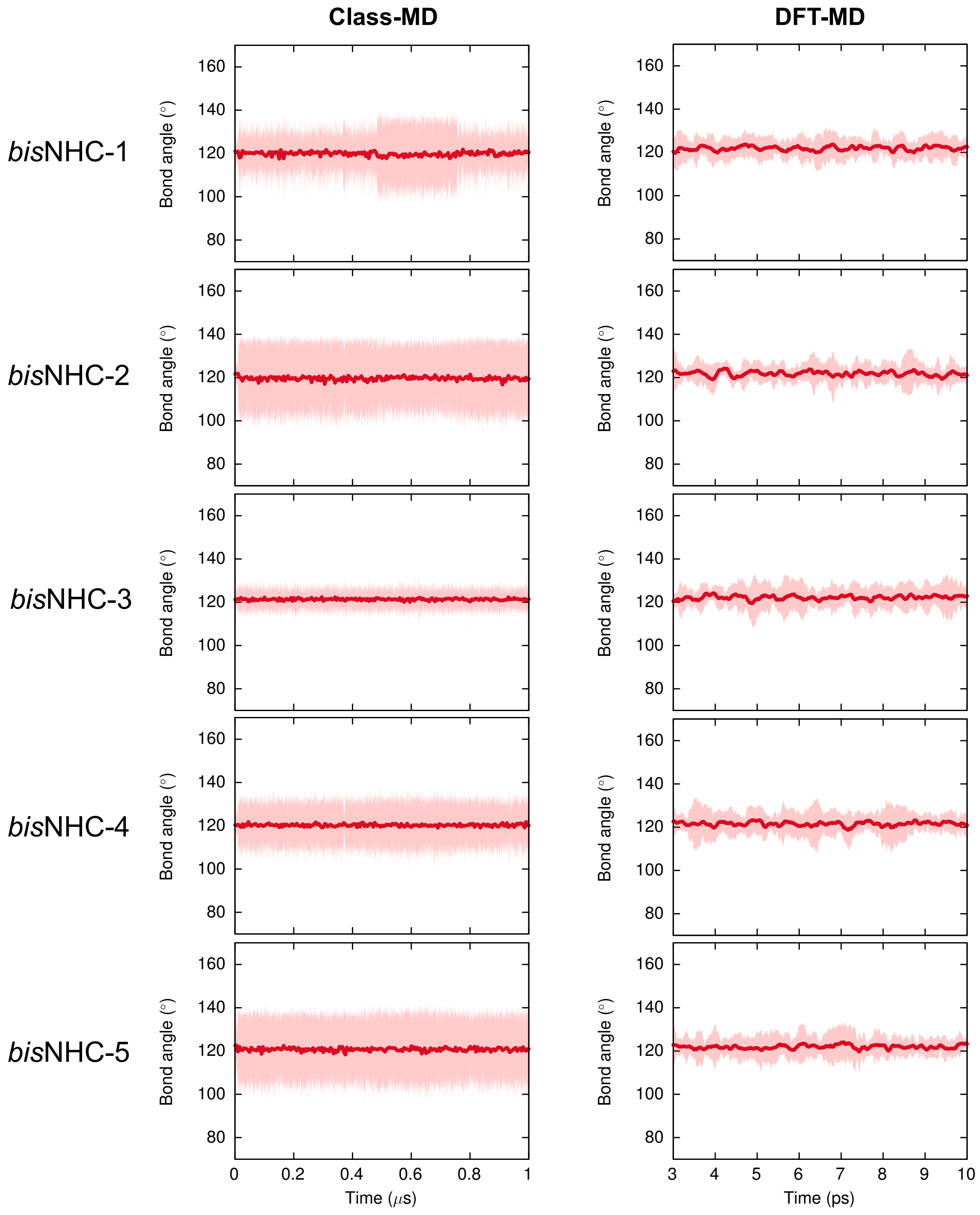

Figure S7. Fluctuation of Au−Au−C angle as a function of time during the MD simulations of 2-Au_{13} nanocluster. Each plot shows the mean bond angle (thick line) ± standard deviation (shaded area) of 10 measurements per frame.

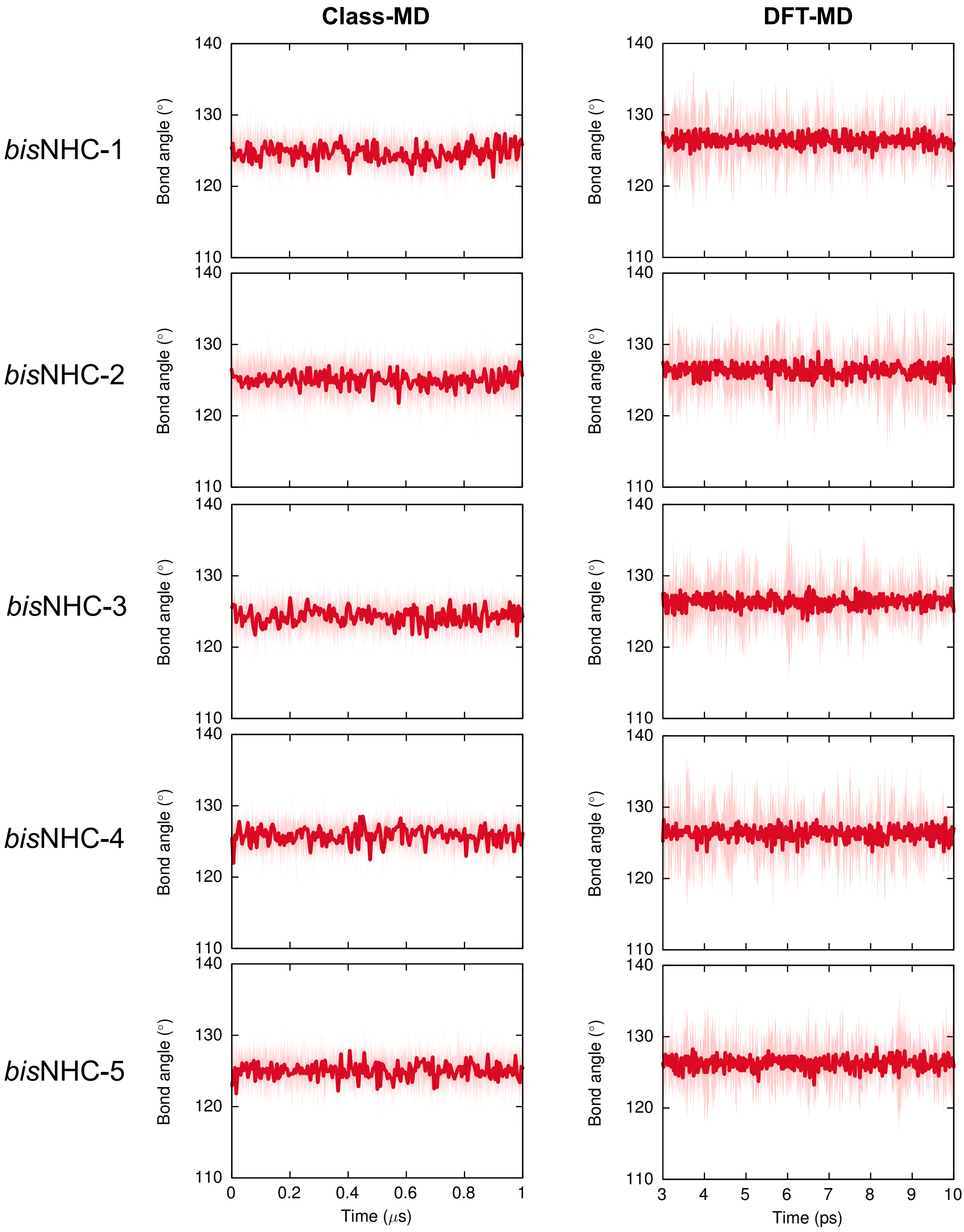

Figure S8. Fluctuation of Au−C−N angle as a function of time during the MD simulations of 2-Au_{13} nanocluster. Each plot shows the mean bond angle (thick line) ± standard deviation (shaded area) of 4 measurements per frame.

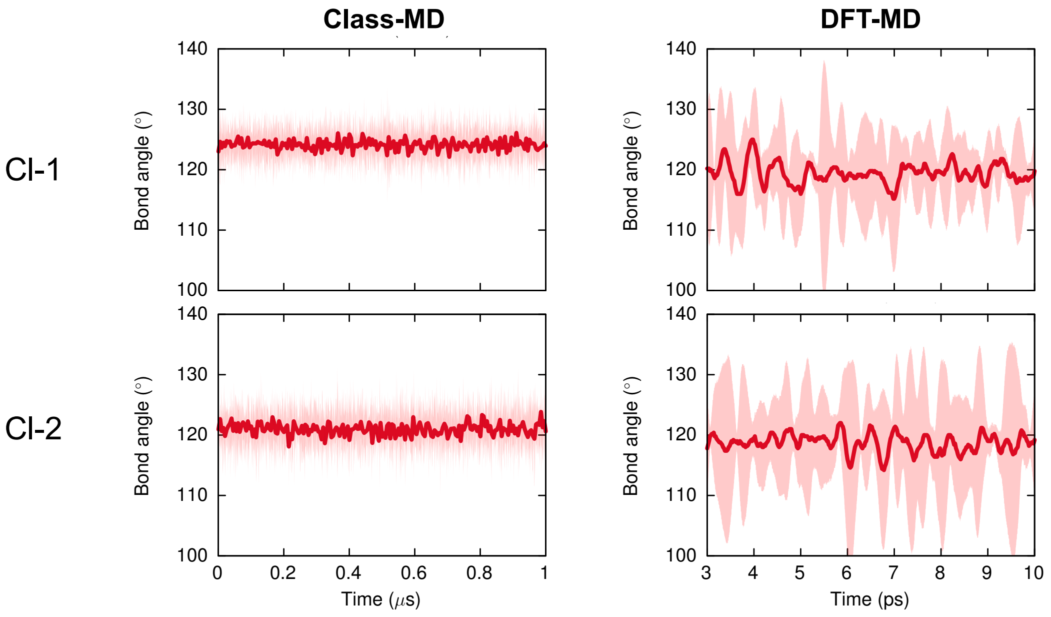

Figure S9. Fluctuation of Au−Au−Cl angle as a function of time during the MD simulations of 2-Au_{13} nanocluster. Each plot shows the mean bond angle (thick line) ± standard deviation (shaded area) of 5 measurements per frame.

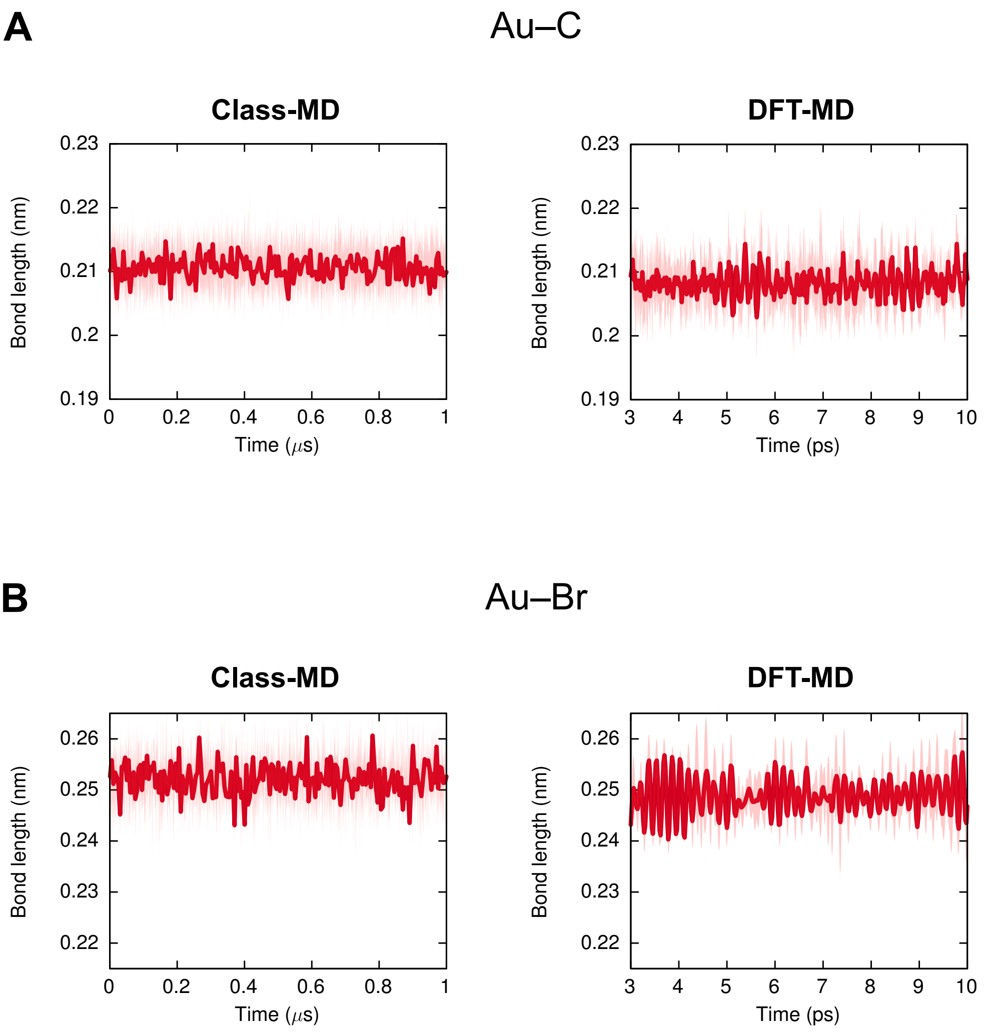

Figure S10. Fluctuation of Au−C and Au−Br bond lengths as a function of time during the MD simulations of 7-Au_{10} nanocluster. (A) Each plot shows the mean bond length (thick line) ± standard deviation (shaded area) of 6 measurements per frame. (B) Each plot shows the mean bond length (thick line) ± standard deviation (shaded area) of 3 measurements per frame.

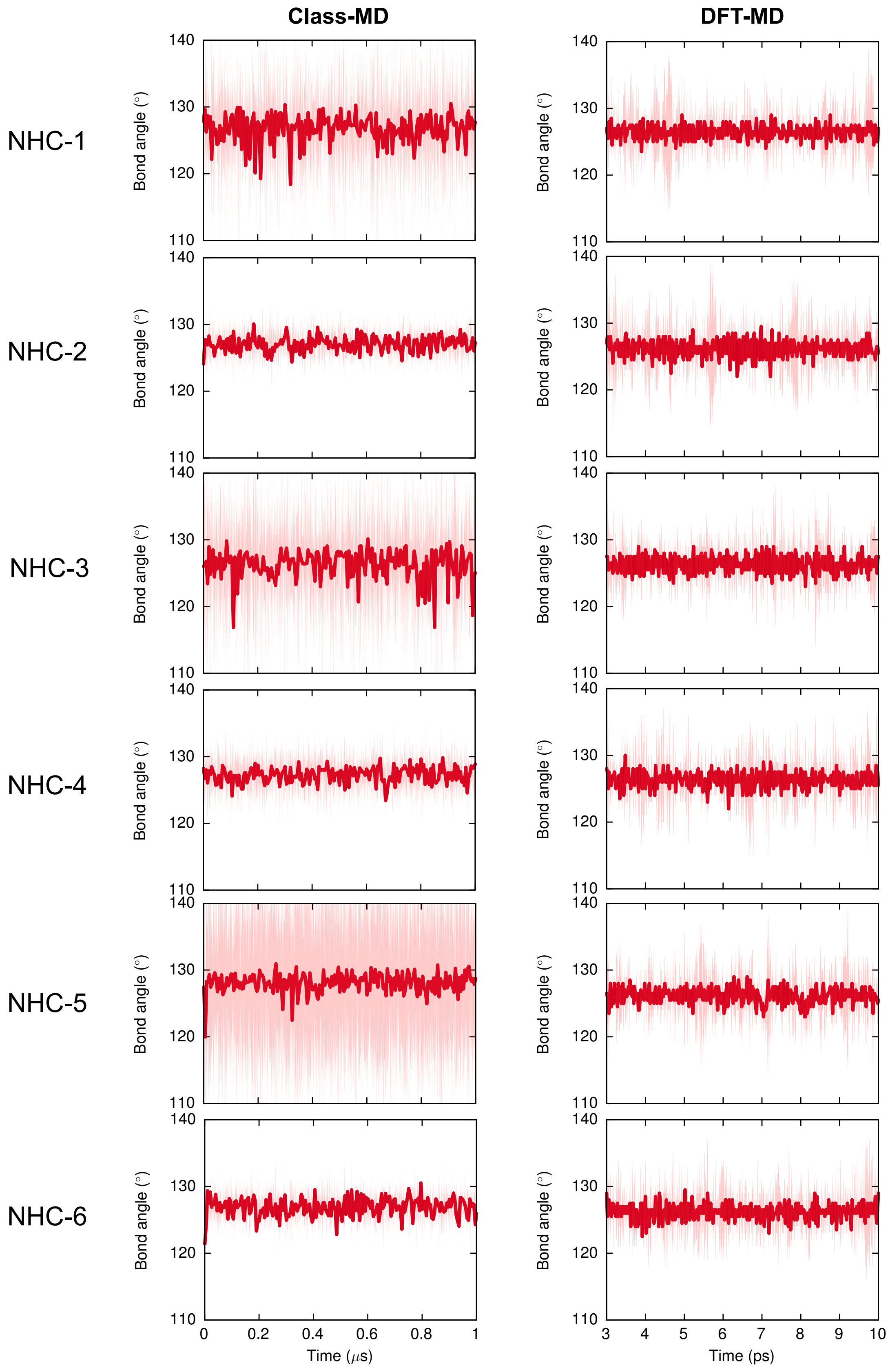

Figure S11. Fluctuation of Au−C−N angle as a function of time during the MD simulations of 7-Au$_{10}$ nanocluster. Each plot shows the mean bond angle (thick line) ± standard deviation (shaded area) of 2 measurements per frame.

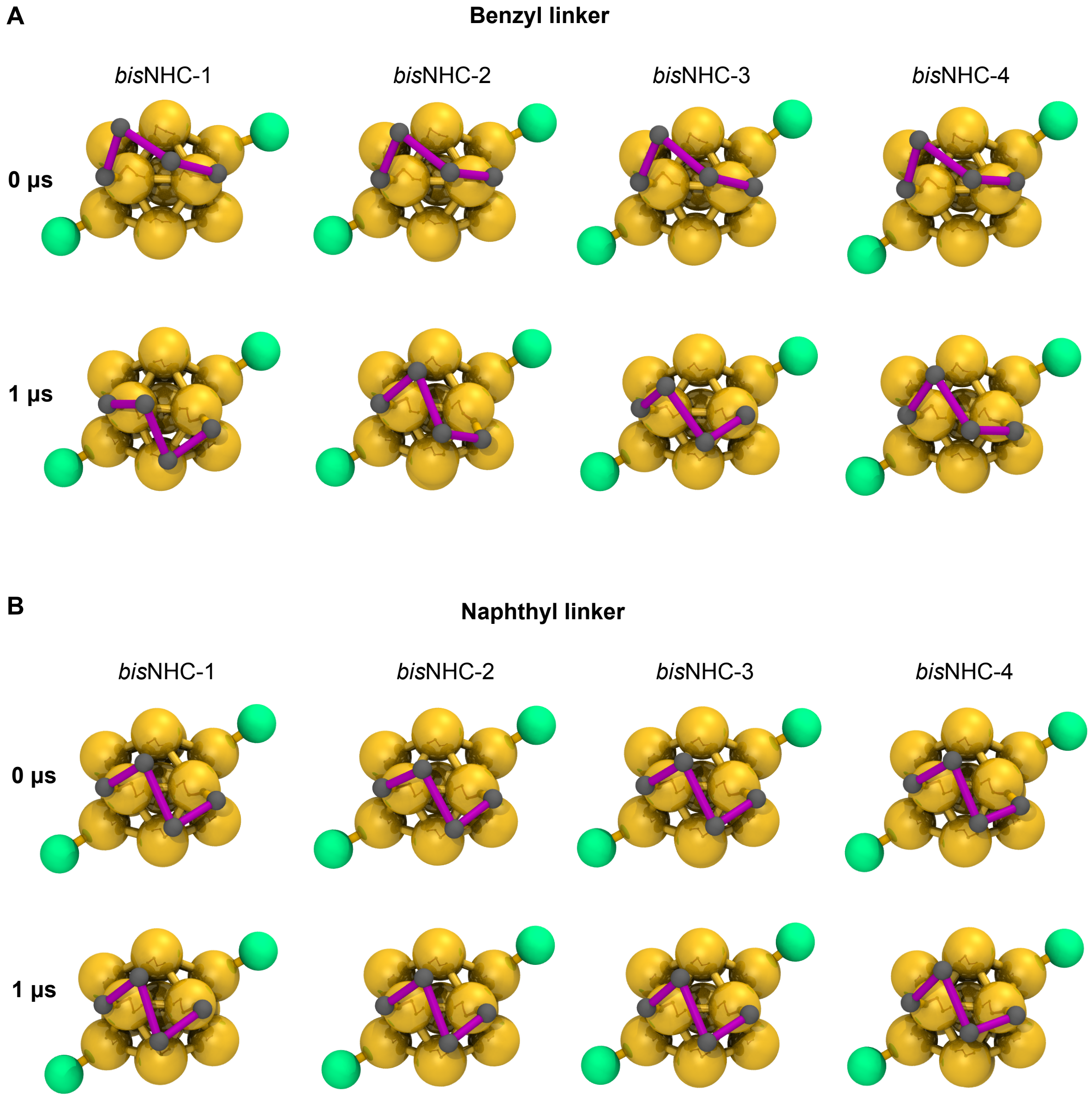

Figure S12. Snapshots from classical MD simulations illustrating the dynamics of C^{Au}–C^{bridge}–C^{Au}–C^{bridge} dihedral angle of *bis*NHC ligands in 2-Au13 and 3-Au13 nanoclusters. Snapshots at the beginning (0 μs) and end (1 μs) of the simulation are shown for (A) *bis*NHC ligands with a benzyl linker in the **2-Au$_{13}$** nanocluster and (B) *bis*NHC ligands with a naphthyl linker in the **3-Au$_{13}$** nanocluster. Color code: Au in gold, Cl^- in green, C in grey. The highlighted purple sticks trace four consecutive carbon atoms within the linker, which define the dihedral angle used to describe internal torsional dynamics.

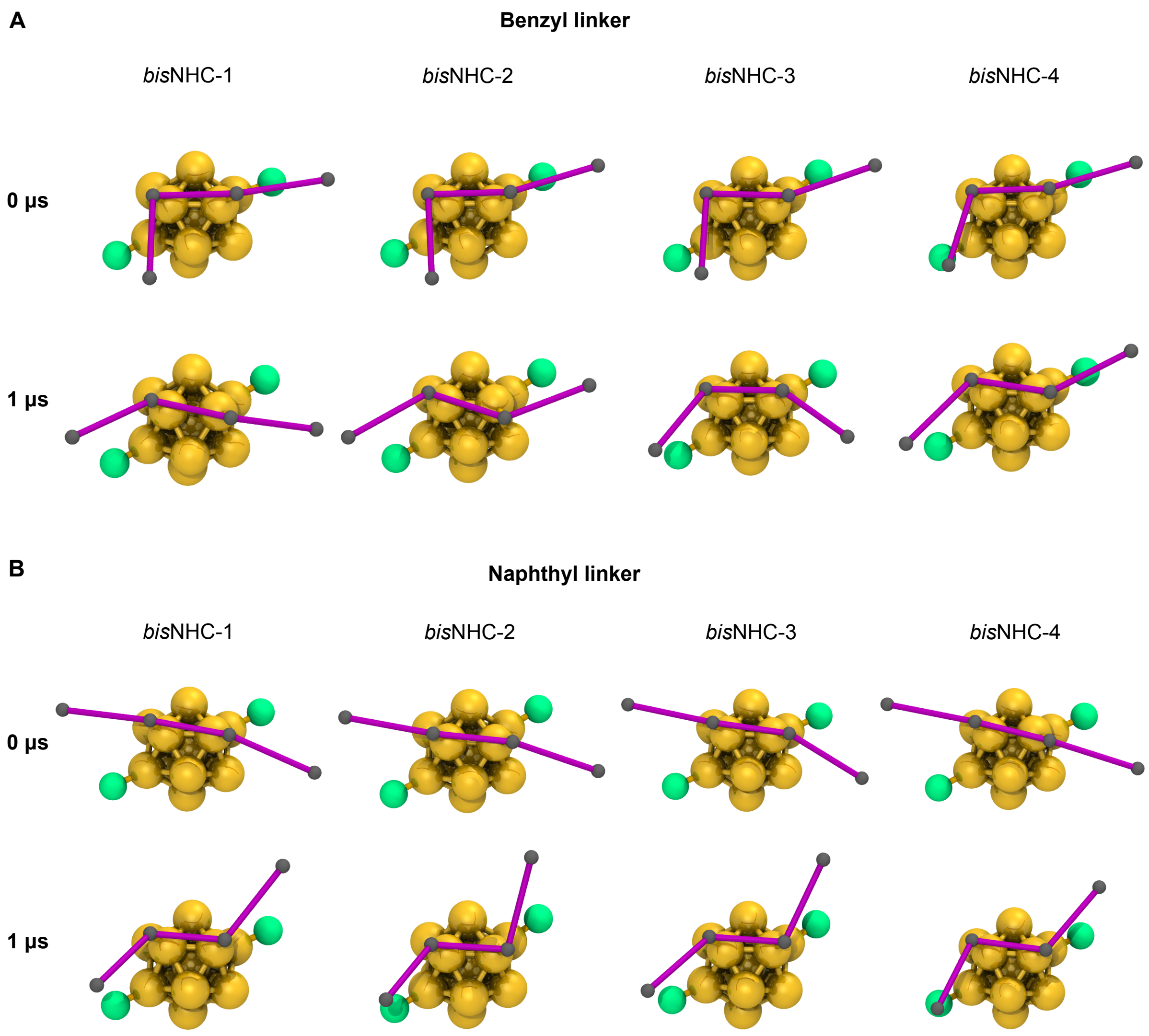

Figure S13. Snapshots from classical MD simulations illustrating the dynamics of C^{wing}–C^{Au}–C^{Au}–C^{wing} dihedral angle of *bis*NHC ligands in 2-Au13 and 3-Au13 nanoclusters. Snapshots at the beginning (0 μs) and end (1 μs) of the simulation are shown for (A) *bis*NHC ligands with a benzyl linker in the **2-Au_{13}** nanocluster and (B) *bis*NHC ligands with a naphthyl linker in the **3-Au_{13}** nanocluster. Color code: Au in gold, Cl^- in green, C in grey. The highlighted purple sticks trace four consecutive carbon atoms within the linker, which define the dihedral angle used to describe internal torsional dynamics.
